# Modeling the Disorder of Closed System by Multi-Agent Based Simulation

**DOI:** 10.3390/e21111105

**Published:** 2019-11-12

**Authors:** Krzysztof Małecki, Tomasz M. Gwizdałła, Paweł Bieńko

**Affiliations:** 1 Faculty of Computer Science, West Pomeranian University of Technology, 70-310 Szczecin, Poland; PawelBienko@live.com; 2 Faculty of Physics and Applied Informatics, University of Lodz, 90-136 Łódź, Poland; tomasz.gwizdalla@uni.lodz.pl

**Keywords:** agent-based modeling (ABM), closed system, complex system, computer simulation, disorder modeling, entropy, multiagent system

## Abstract

Mess (disorder)—there are many different meanings related to this problem. The explicit majority comes from the area of philosophical, social and medical sciences. In our paper, we try to present the engineering aspect of the concept of disorder. We propose a mathematical model which describes the effects and consequences concerning the process of making the mess. We use Multi-Agent Modeling, where there are several independent agents with decision-making ability. Each agent has the ability to communicate and perceive for achieving its own aim. We use square grid *n* × *n* with objects which can be moved by agents to another places. The degree of disorder of the system is examined by the value of entropy. Using computer simulation, we investigate the time needed to find the desired thing in an environment in which agents (in real life, people) co-exist and they have different tendencies to tidiness. The cost of mess is counted as the number of attempts to access the object in the analyzed system and the time needed to locate the object.

## 1. Introduction

The concept of order finds its beginning in philosophical, systemic and technical works. The French philosopher Bergson [1] described order as a being that facilitates humankind’s functioning in the world. Because of this being, humankind creates patterns of action, which are the basis for realizing its practical goals. In turn, in the theory of disorganization and disorganizing behavior [2], the notion of disorder (mess) as the unorganized accumulation of various objects was defined. A disorder can be caused by both individuals and groups of participants (agents). We can talk about a mess in the system when there the following is present [2]:

- disorder—a deviation from hierarchical order; when one or more agents are unsuccessfully trying to create a hierarchical order (the deviation can be either intentional or unintentional);
- accumulation—the creation of disorder as a result of cumulation has a temporal dimension. Messes can result from distinct processes, evolve at different speeds and persist for varying durations. Messes can occur relatively suddenly when an exogenous shock destroys the hierarchical order. However, messes can accumulate relatively slowly, as a result of messiness (e.g., putting things back elsewhere); and
- a differentiation of objects—when there is no possibility of categorizing objects.

The attempt to organize a mess is called creating order. This process is complex and time-consuming. Even if every participant (agent) creating order is guided by hierarchical categorization and a certain organizational scheme, there is still the risk of creating a mess [2,3]. Each participant (agent) organizing the order according to his/her own rules, without prior arrangement of the order style, as a consequence, generates a mess.

A source of inspiration on messes is complexity theory [4]. It examines entropy and the emergence of order out of disorder. Further studies [5,6,7] provide insight on some of the benefits and consequences of certain types of messes. Order, disorder (mess) and hierarchy are introduced from certain perspectives—economic [8], political [9], sociocultural [10,11] and others [12]. A mess (the subject of this article) may occur at various levels of analysis (at the level of the economy, the industry or the organization), in different types of systems (individual, collective or formally organized) and in various locations (room, file, servers with data or government). The permanent observation of all areas and complex systems seems impossible.

Hence, modeling is a necessary mechanism for understanding complex phenomena [13,14] such as messes [15]. We use models to gain an understanding of complex phenomena; indeed, modeling is “a purposeful abstraction of reality” [16]. Reference [17] states, “a model should be a close approximation to the real system and incorporate most of its salient features. On the other hand, it should not be so complex that it is impossible to understand and experiment with it. A good model is a judicious trade-off between realism and simplicity”. It is a necessary simplification of the real-world system it models.

In the article, we consider an isolated, closed system composed of noninteracting identical particles. The initial, hierarchical state of the system can be changed by agents interfering in the system order by changing the position of individual particles. Based on the changes made, we calculate the entropy value of the system and other indicators (access time and amount of access and effort), providing the opportunity to assess how much the order of the system has been disturbed and what consequences it brings for individual agents. In other words, we reflect the real situation associated with a certain room, in which there are some objects whose location can be changed by certain people. Changing the position of an object affects the time it takes to find the object and the effort associated with finding it. In fact, this translates into a waste of time, for example, an employee looking for a document that has not been put back to its original place.

Entropy [18] is understood as a measure of disorder [2]. The observation of patterns in nature prompts the asking of questions about the origin of entropy. Understanding this phenomenon and the role it plays in systemic thinking requires recalling the second law of thermodynamics, where the entropy (the property of matter at the microscopic level) can be created but it can never be destroyed.

We deal with the concept of entropy every day. It is nothing else but a measure of the disorder of the system—a mess that comes from interfering in the initial order. To answer the question as to what the actual cause of the mess is, it would be necessary to carry out appropriate research, involving people who would perform the changing of the place of different objects. It should be noted at this point that the completion of such research in a reasonable time would be determined by the rapid performance of instructions by those employed in the research, which seems impossible to do. Fortunately, modern technology allows us to conduct this type of research using computer simulation [19] and multiagent systems [20,21,22].

To answer the question about the impact of agents using resources on the creation of entropy, it is necessary to use sociological [23] and cognitive sciences [24]. The analysis of several theories related to this topic, including the theory of disorganization, the issue of cognitive science in connection to game theory [25], which systematized resolving conflicts over access to resources, allows for an understanding of the problem and the simulation of this phenomenon.

According to Reference [8], ordered systems are desirable systems. Therefore, the purpose of this article is to check the unfavorable effects of the disorder of a closed system in the context of time, effort and increasing entropy. To achieve the goal, we developed a mathematical model of the studied phenomenon and implemented it in the form of a multiagent system that allows for conducting research on various potential agents’ behaviors. For our task, we have put forward the thesis that different agents’ strategies of access to objects are the particles of the closed system that cause a diverse disorganization of the system, which we can estimate using entropy values, as well as other parameters such as the number of accesses to objects, access time to the facility and effort. In the subsequent part of the article, we define all these measures.

The article is organized as follows. In Section 2, we include the related works. Section 3 presents a theoretical background (the entropy calculation, description of the terms we have used and the description of the model). Then, the experimental results and the discussion are shown. The last section describes the conclusion.

## 2. Related Works

The selection of articles available in the scopus.com database allowed us for analysis of studies related to multiagent modeling. The database has been questioned in relation to the following keywords—multiagent, mess and disorder. 152 results were obtained. The vast majority concerned only one of the listed phrases and they are not at all connected to presented approach. Another group of results is a group of medical articles, in which disorder is the word determining the organization of cells (e.g., skin, blood) of a specific case of disease.

Only a few studies were strictly related to multi-agent simulation in the aspect of the disorder factor. For example, in Reference [26], the transfer of the messages in large distributed multi-agent systems is studied. The authors have proposed the method of messages transfer through linking events. The results show an efficient transmission of messages between agents, including extract associated events from mess messages. In the aspect of manufacturing execution an interaction mechanism designed around the concept of order and resource agents implementing the monitor-analyze-plan-execution loop have been described in Reference [27]. In Reference [28] the authors investigate the multiagent evolutionary games on a lattice and their extension by allowing the players to use additional neutral strategies that provide zero payoffs for both players if one of them selects one of the neutral strategies. They conclude that in the resulting *n*-strategy evolutionary games the analytical methods and numerical simulations indicate continuous order-disorder phase transitions when increasing the noise level if *n* does not exceed a threshold value. In Reference [29] authors have investigated how the frustration of a social network influences the appearance of nonzero equilibria as a function of a scalar parameter playing the role of social effort. In Reference [30] authors used the Lefebvre’s “algebra of conscience” to describe decision-making strategies of agents simulating people with different brain dominance. They suggest that the emergence of the two principal statistical distributions is able to illustrate different types of society organization and also to be used in order to simulate market phenomena and psychic disorders, when a switching of hemisphere dominance is involved.

The disorder can be also considered as social disorder generated by traffic congestion such as delays, economic losses and environmental pollution in urban life. Using multiagent systems such disorders are investigated for example in References [31,32,33].

In our approach, we rely on the concept of an “actor” defined by Hewitt [34], which is defined as a self-contained, interactive and concurrently executing object which has some encapsulated internal state and could respond to messages from other similar objects. As Hewitt stated, an actor “is a computational agent which has a mail address and a behavior. Actors communicate by message-passing and carry out their actions concurrently”. Wooldridge defined a deliberative agent as “one that possesses an explicitly represented, symbolic model of the world and in which decisions (for example about what actions to perform) are made via symbolic reasoning” [35].

Based on the experiences from previous studies, we propose a model that allows interaction between agents based on the shifts of objects available on the *n* × *n* board. This is a kind of game where agents perform specific tasks without assessing the profit and loss for each of them. The leitmotif is to check the parameters of the system, that is, entropy, the level of system disturbance and the time needed to organize the system. Agents reflect people in the real world, particles on the board mean objects that surround us, shifting the particles means the process of using real-life objects and putting them back to the original place or to another, random place. The whole scheme reflects the coexistence of people (e.g., in project team) who create a mess in the common space, making the task handled by companions more difficult to perform.

## 3. Proposed Approach

### 3.1. Entropy Calculation

To determine the measure of disorder, let us briefly recall the basics of the notion of entropy and its application to the system under study. Throughout the paper, we use the common Shannon entropy:

(1) $$S=-\sum_{i=1}^{n}p_{i}\log\left(p_{i}\right).$$

with $p_{i}$ being the probability of particular state. One can certainly expect that some more sophisticated form of entropy, such as the generalized Renyi [36] entropy, could be used. Two factors, however, in our opinion, motivate our choice. First, the simplicity of the Shannon formula and its generality make it possible to more easily familiarize the results. Second, the selection of the α parameter (alpha corresponds to the exponent of probability used in summation) for the Renyi formula would necessitate a detailed discussion in the context of the studied problem. We can also show that often the studies of extended propositions of entropies are considered as particular problems, such as in References [37,38].

The symbols we use in further considerations are as follows:

- *N*— the number of positions available for objects,
- *n*—the number of types of objects and
- $n_{i}$, i=1…n—the number of objects of a particular type.

Additionally, the empty positions have to be considered in statistical calculations as a separate (n+1)th type of object. The number of empty places is certainly given as follows:

(2) $$n_{e}=N-\sum_{i=1}^{n}n_{i},$$

where the summation is taken over all possible configurations of system.

The basic assumption when taking into account the particular objects is that about their nondiscernability or even identity. The problem can be discussed at different levels of generality. The crucial remark, however, is that the set of the object’s features that are available to the observer follow the Leibniz principle of identity. Therefore, the number of possible realizations of the system equals the number of permutations with repetitions:

(3) $$k=\frac{N!}{n_{e}!\cdot \prod n_{i}!}=\frac{N!}{\left(N-\sum n_{i}\right)!\cdot \prod n_{i}!}$$

In this paragraph we consequently denote—the number of configurations by *k*, their probabilities by *p* and entropies by *S*; the index corr corresponds to the correct configuration while ncorr to the all incorrect ones.

Among the *k* arrangements given by Equation (3) certainly $k_{\mathrm{corr}}=1$ is correct, so the probability equals 1 and the entropy $S_{\mathrm{corr}}=0$. To determine the entropy of the disordered state, we have to make some additional assumptions. In the initial phase we have no knowledge about any position of an object, so we cannot reduce the remaining number of k−1 configurations. The exact value of entropy depends here on the likelihood of realization of the particular configuration. Since we have no suggestion about the preference of different patterns, we can assume that every configuration is equally probable and this assumption will be used throughout the whole paper. Therefore, we can write the following for the entropy of the disordered state: $S_{\mathrm{ncorr}}=-\left(k-1\right)\cdot p_{\mathrm{ncorr}}\cdot lg\left(p_{\mathrm{ncorr}}\right)$. Since $p_{\mathrm{ncorr}}=1/\left(k-1\right)$, we can finally write the following:

(4) $$S_{\mathrm{ncorr}}=-lg\left(p_{\mathrm{ncorr}}\right)=-lg\left(\frac{1}{k-1}\right).$$

The situation presented above is certainly well known and can be, on the other hand, considered an extreme case. In reality, we have some knowledge about the system that reveals the fact that we can enlist some objects as occupying the appropriate positions. For the localization of other objects, we can assume, however, one of two statements:

- We either have no information about other objects or we are not interested in them. This leads to the conclusion that some of the objects not enumerated during the procedure described above can occupy their correct position. For some reason, we do not have this information.
- We are sure that no one from other objects is located in an appropriate position.

We can imagine both situations as follows—the first one corresponds to the covering of the whole board of the game with some overlay, while the second one corresponds to the walkthrough of all positions with the expected presence of some objects with a negative result. The choice of one of the presented models strongly influences the results due to the differences in the amount of information provided by both methods. Our knowledge about the system is much greater when using the second approach compared to the first one. Certainly, the second approach restricts much more the entropy value and we decided to use further the first one.

We additionally have to define two notions related to the occupied positions. Let the following hold:

- M—the number of positions that are occupied by the correct, presumed object and
- $m_{i}$, i=1…n—the number of objects of a particular type in the correct positions.

Certainly, $\sum_{i=1}^{n}m_{i}=M$.

The crucial observation is now the one in which we have exactly the same question as earlier in this section. We must simply distribute $\sum (n_{i}-m_{i})$ objects in N−M positions. Therefore, once more, the permutations with repetitions are the solution to the problem and the appropriate values can be calculated as follows. The number of configurations (3) corresponding to the previous formula are as follows:

(5) $$k_{1}=\frac{(N-M)!}{\left(n_{e}-m_{e}\right)!\cdot \prod (n_{i}-m_{i})!}.$$

In addition, the entropy is simply the modification of formula (4) using the above value of $k_{1}$:

(6) $$S_{1}=-\mathrm{lg}\left(p_{k_{1}}\right)=-\mathrm{lg}\left(\frac{1}{k_{1}-1}\right),$$

where $n_{e}$ is the number of correctly unoccupied (empty) places and among the $m_{e}$ which should be unoccupied. We use the index 1 just to distinguish this case from the previously described one.

### 3.2. Descriptions of the Terms Used

**Figure 1:**
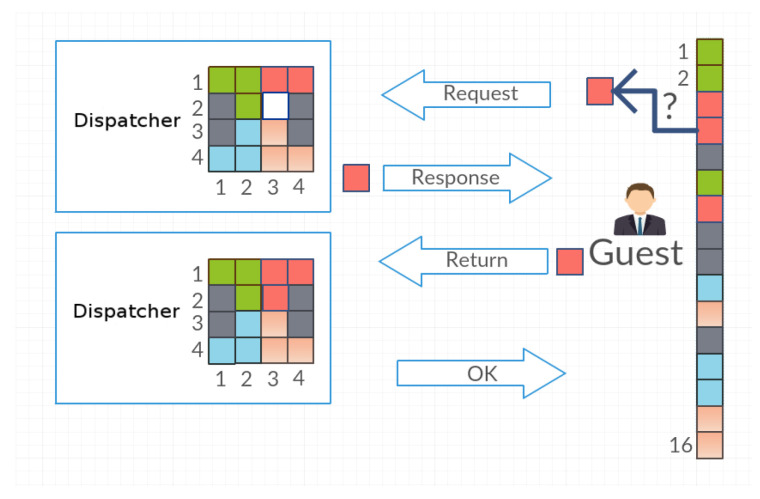
The interaction between Dispatcher and Guest. Dispatcher manages the matrix of objects—gray squares symbolize the free space in which Guest agents can postpone downloaded objects, colored squares are objects of various types. The column of the colored squares at the Guest agent symbolizes the register of object positions in the matrix managed by the Dispatcher agent (square number 1 is the object on position [1, 1] in the Dispatcher matrix, and so on).

In this section, we present a few terms used in the article for better understanding the model and discussion:

- The system (the closed system)—a finite set of objects whose location will change depending on the requests of agents of the multiagent system.
- The object—one of the elements of the closed system. The number of objects in the system can be any number. For example, for a 4 × 4 grid, the maximum number of objects is 16.
- Dispatcher—the agent managing the set of objects. This agent receives requests to access the selected object by agents: Guest 1, Guest 2 and Guest *n*. If the Guest agents request access to the same object, the Dispatcher grants access to the object depending on the strategy being considered—randomly or in accordance with the principles of game theory.
- Guest—one of many agents in a multiagent system who is trying to access objects in the system. Before starting the simulation, he/she writes to his/her own register the information about the layout of objects (objects’ positions in the matrix) because, in the beginning, his/her objects exist only in the Dispatcher matrix. The Guest agent, after he/she receives the object, may postpone it to the same place or place it in a different place with the probability level set for him/her. This process is called the disorganization of the system or the generation of a mess. The Guest agent requests the object from the Dispatcher in accordance with the tactics used—in the order of agents or in accordance with the tactics of game theory.
- The interaction between Dispatcher and Guest—a Guest agent selects from the register the coordinates of the object that he/she is trying to access and then sends it to the Dispatcher with the request to release the object (Figure 1). In response to the request, the Dispatcher sends the object, marking its place as a blank. Once the Guest finishes using the object, he/she sends it back with the coordinates of the place in which he/she wants to place the object. The Dispatcher saves the object in accordance with the transferred coordinates and then terminates the communication with the Guest agent by replying OK. Such a series of actions is a part of the effort of the Guest and it is equal to 2 [Request, Return]. The time when one application proceeds (e.g., Request) is called the time of the agent’s access to the object.
- Effort—the number of attempts to access a given object regardless of what action the Guest agent performs. One cycle associated with picking up and returning an object yields an effort equal to 2.
- The time of the agent’s access—the access time to the expected object, from the moment the Guest sends requests to the Dispatcher until he/she receives the object.
- Entropy—the measure of the system disorder.

### 3.3. Multiagent Model

Wooldrige [39] has defined the concept of an agent as “an independent computer system, placed in a specific environment, able to undertake autonomous actions and decisions in order to implement specific tasks”. Huhns and Singh [40] have defined this concept as “a computer entity capable of reacting, inferring, initiating action and communication”.

A multiagent system is a system consisting of many independent, albeit cooperating, agents. This is what distinguishes a multiagent system from other distributed systems, wherein the large autonomy and activity of individual elements, as well as the openness of the architecture, is obtained because of the standardization of communication. Multiagent systems enable the construction of diverse applications, which are easily scalable and transformable. The standards presented by the FIPA organization document all the relevant patterns of the multiagent system, including the communication of agents and languages of the message description (metalanguage), agent management, the structure of services and elements of the multiagent system. Normative architecture has been defined in documents collected under the FIPA Abstract Architecture. It standardizes the basic elements of the system—communication mechanisms, system management tools, services and requirements for the agent’s interface.

**Figure 2:**
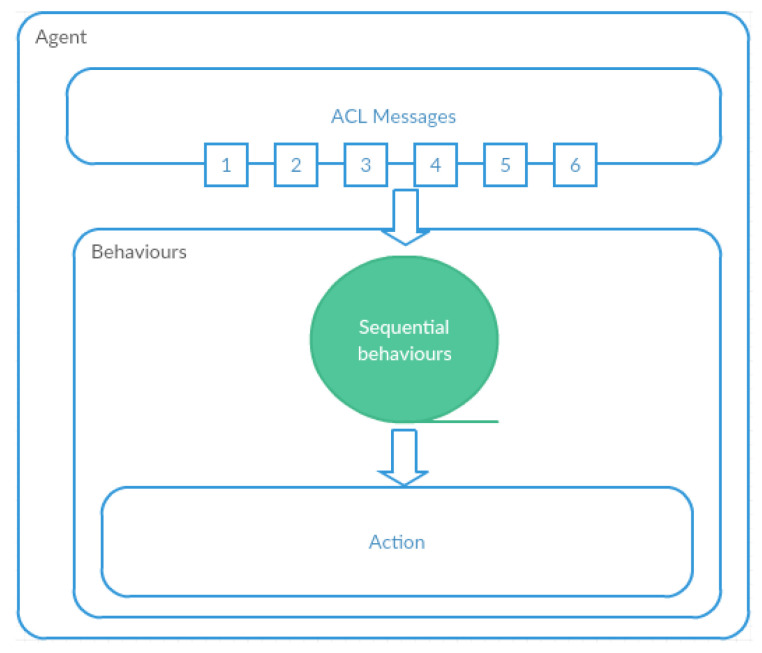
The agent architecture based on behaviors.

We have implemented a multiagent system based on the JADE (Java Agent Development Framework) platform, which is compliant with the FIPA standard, characterized by open source, code and is made available under the LGPL license. The concurrent agent functions were performed using behaviors (Figure 2). The behaviors are used to control complicated, sequential agent work processes. Concurrency is performed on the basis of the cyclic activation of a given behavior, one after the other, which allows for the solving of the problem of synchronizing access to data.

**Figure 3:**
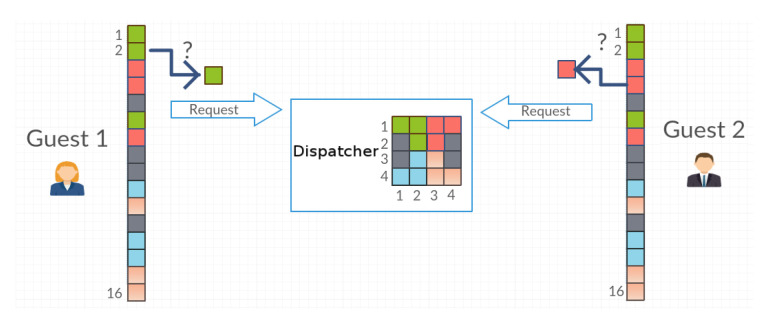
Agents send a request for objects.

Let us denote agents as $A_{1},A_{2},\ldots ,A_{n}$ and the set of objects as $O=o_{1},\ldots ,o_{n}$. In the unit of time *t*, any agent can possess only one unique object $o_{i}\in O$. Agents have knowledge about the location of objects compatible with the collection *G* of the Dispatcher. Guest agents synchronously or asynchronously request a specific object for the resource manager (Dispatcher agent) (Figure 3). For the sake of the simplicity of further reasoning, two agents, $A_{1}$ and $A_{2}$, were assumed to be labeled as Guest1 and Guest2, respectively.

**Figure 4:**
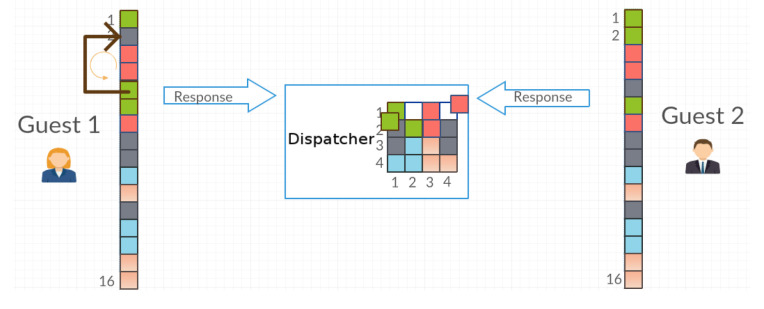
Agents give back the objects. Guest1 has exchanged object 2 with 4.

The Guest agent receives the requested object and, after its use, returns the object in the place originally assigned to it $pos_{i}\in G$ or in a random place with the probability *P* from a set of free locations $G_{empty}$. If the Guest agent places the object in a different location, it records the new location of the object in its memory (Figure 4).

**Figure 5:**
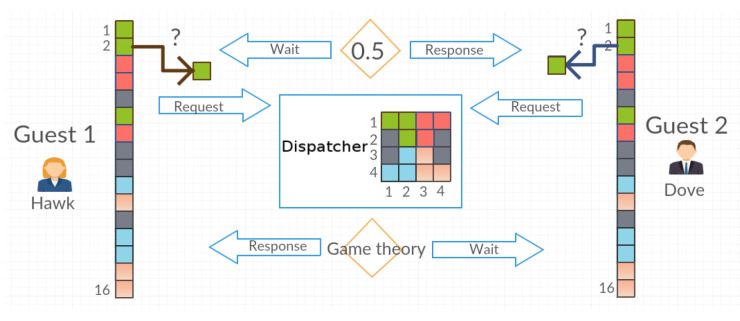
Dispatcher sends objects in accordance to tactic.

In the situation where both agents request access to the same object, to whom it will be assigned can be selected in two ways. The first method is to randomly select one of the agents with the probability of getting access P=0.5. The second method consists of selecting an agent in accordance with the tactics of game theory (hawk and pigeon model). If both agents have the same tactic (both are hawks or both are pigeons), the probability of winning is the same, P=0.5. Implementing the hawk tactics, each agent loses 0.5 seconds for combat, while 2 seconds for pigeon tactics. An agent that does not receive the requested object in a given queue, in the next iteration, requests the object again (Figure 5).

**Figure 6:**
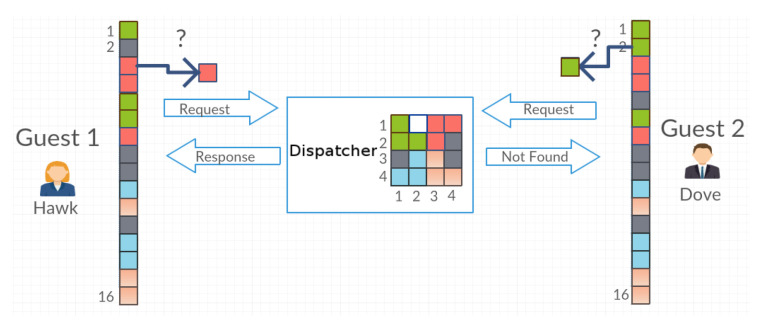
Agent Guest 2, requested the access to the object, which in the previous moment of time was postponed by the Guest 1 agent to a different place. It receives the “Not Found” message from the Dispatcher agent.

**Figure 7:**
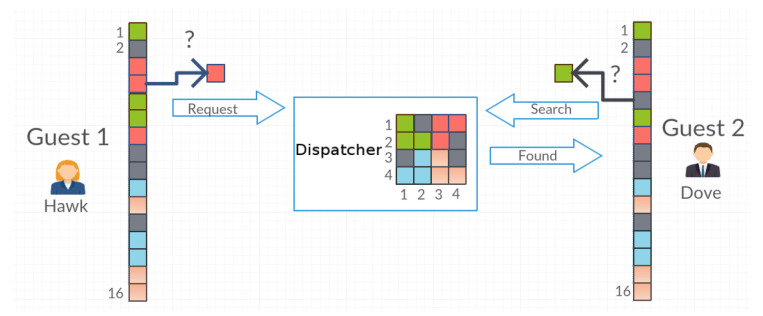
The Guest 2 agent reports the next possible coordinates of the searched object. When it hits the right one, it receives the “Found” message from Dispatcher.

If there is a request to issue an object that has been moved to a place other than its original one, then the process of searching for the object takes place. The Guest agent starts this process from the first to the last object in its register. At the same time, it stores in its registry the current arrangement of objects from the matrix belonging to the Dispatcher agent (Figure 6 and Figure 7).

**Figure 8:**
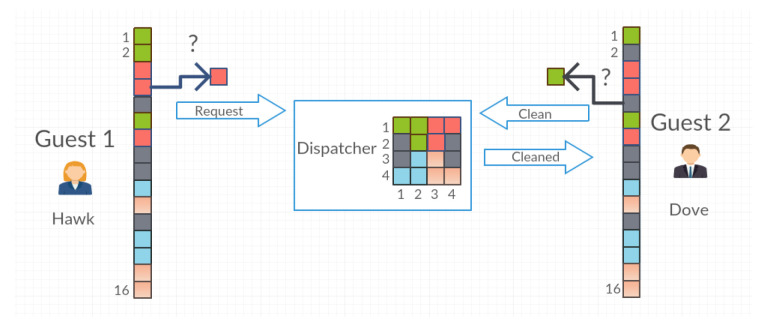
The situation of cleaning the system by the Guest 2 agent.

Agents can also work in cleaning mode. In this mode, the Guest agent finds in the registry the objects postponed by him/her in a place other than the initial one and then sends them to the Dispatcher agent with the “Clean” action. The Dispatcher agent, based on the initial coordinates, places the object in the originally assigned place and then responds to the Guest agent with the message “Cleaned” (Figure 8).

It is worth noting that agents do not act in isolation. Agents perform operations on shared objects, and even if the agent knows where he put down the object, he does not know what the other agent did.

### 3.4. Model Validation

**Figure 9:**
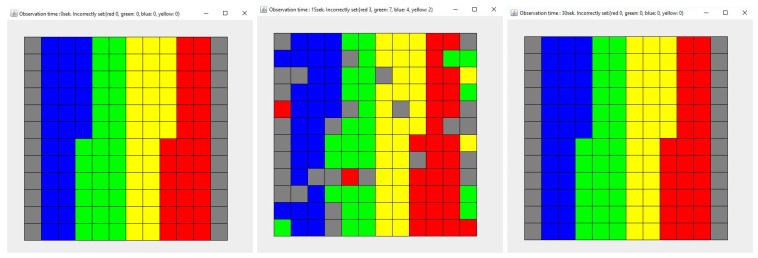
The state of the system before starting the simulation (**left**), after the first simulation stage (**middle**) and after the second simulation stage (**right**). Each color describes the type of elements. Here are four types of elements and each type consists of 30 elements.

To validate the model, a simulation consisting of two stages was performed—(1) disorganization of the system by two agents and (2) cleaning the system. Figure 9 shows the layout at the beginning of the simulation, after the first stage and after the second stage of the simulation. During the first stage, each of the agents requested 50 times from the Dispatcher to access the objects, assuming that Guest 1 places objects in their original location and that Guest 2 places objects in random, empty places with a probability of 0.2. The second stage, cleaning the system, was carried out until the system returned to the initial state (all objects were in their original places). Entropy was used to assess the state of the system and it should have the same value at the beginning and the end of the simulation.

**Figure 10:**
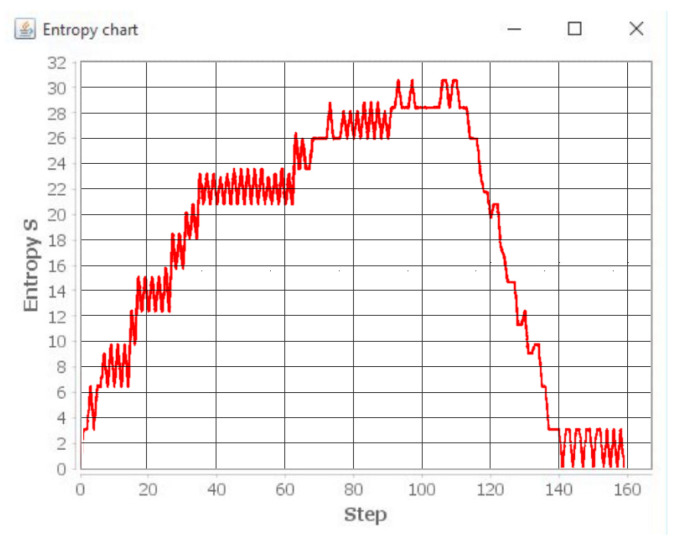
Entropy of the closed system for the simulation with two stages—disordering and cleaning.

The placement of objects in the Dispatcher’s matrix has not changed relative to the initial state. The entropy value of the system in the individual simulation steps is shown in Figure 10.

During the simulation, agents obtained access to objects 158 times. The entropy of the system increased during its disorganization by agents (stage 1). When the cleaning process started (stage 2), the entropy value of the system started to decrease until the initial value equaled 0. An unsteady graph line results from the system operation in the asynchronous mode, in which agents access the objects alternately and entropy is counted after each returned object, so its measurement during the test often falls out when one of the agents has taken the object and has not returned it yet. The first stage lasted 100 iterations, during which the system’s entropy increased up to a maximum value of 30.58. In subsequent iterations, the entropy decreased until it reached the value of 0 at the end of the second stage of model validation process. The system returned to its initial state within 30 seconds. Since the ordering of the system ended with the initial state of the system, it means that the developed model and system work correctly.

**Figure 11:**
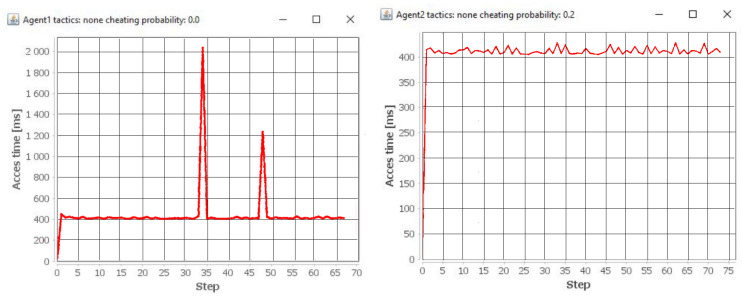
The access time of individual agents for the case of cleaning a temporarily unordered system.

The time of access to objects for both agents is different (Figure 11). This results from the situation where a given agent tries to access an object that has been moved to a location other than the original one.

## 4. Numerical Results

**Figure 12:**
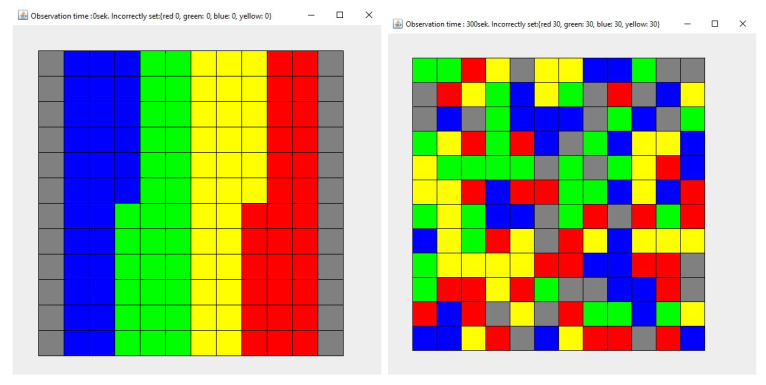
The state of the system for the simulation 1, at the beginning (**left**) and after the simulation (**right**). Each color describes the type of elements. Here are four types of elements and each type consists of 30 elements.

**Figure 13:**
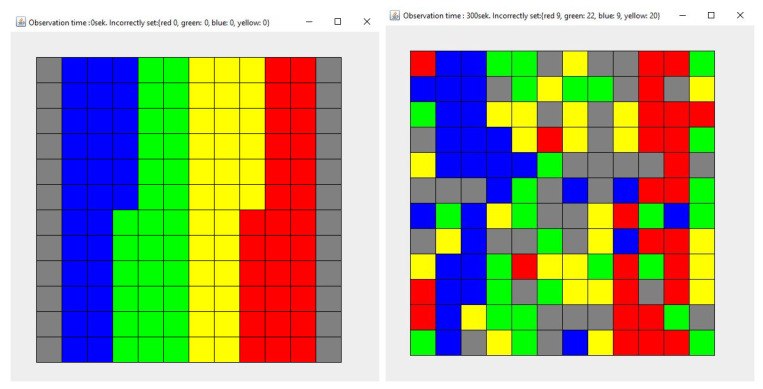
The state of the system for the simulation 2, at the beginning (**left**) and after the simulation (**right**). Each color describes the type of elements. Here are four types of elements and each type consists of 30 elements.

**Figure 14:**
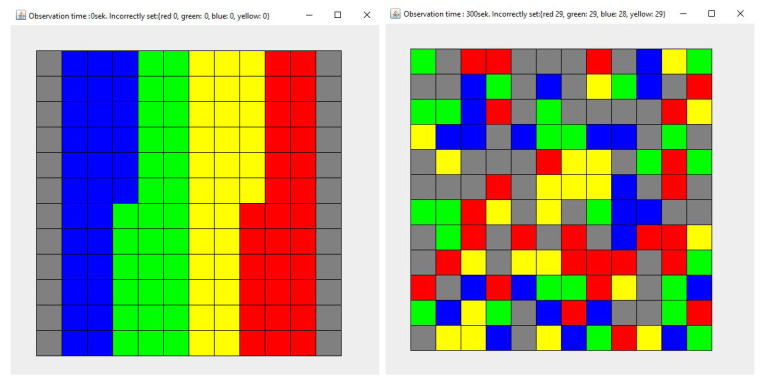
The state of the system for the simulation 3, at the beginning (**left**) and after the simulation (**right**). Each color describes the type of elements. Here are four types of elements and each type consists of 30 elements.

**Figure 15:**
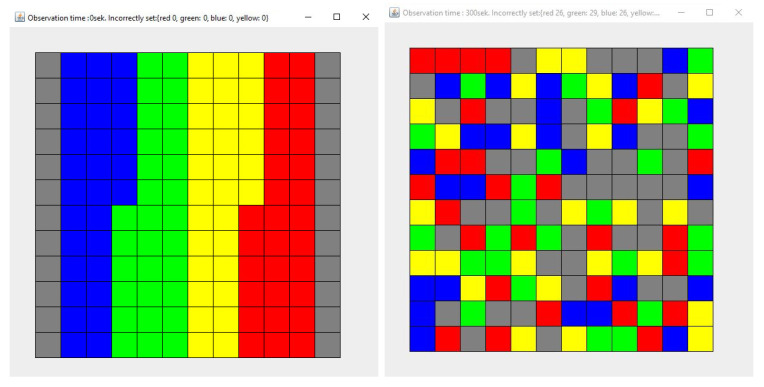
The state of the system for the simulation 4, at the beginning (**left**) and after the simulation (**right**). Each color describes the type of elements. Here are four types of elements and each type consists of 30 elements.

This section presents the results of the simulations, assuming that both agents (1 and 2) place objects in a place other than the original one. Subsequent research concerns various cases:

- Simulation 1—Agent 1 places the collected items in a different place with a probability of 0.9, while Agent 2—with a probability of 0.1 (Figure 12).
- Simulation 2—Agent 1 and Agent 2 place the collected items in a different place with a probability of 0.1 (Figure 13).
- Simulation 3—Agent 1 and Agent 2 place the collected items in a different place with a probability of 0.5 (Figure 14).
- Simulation 4—Agent 1 and Agent 2 place the collected items in a different place with a probability of 0.9 (Figure 15).

The set (0.1, 0.5, 0.9) was chosen as a case of boundaries values and the middle value. The chosen set is enough and reflects the characteristics of the system and the process.

**Figure 16:**
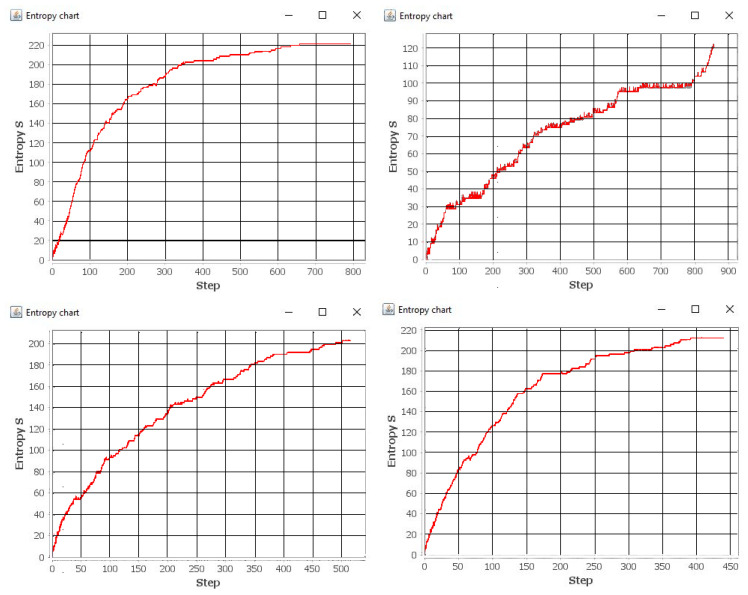
The entropy for the simulation 1 (**top left**), simulation 2 (**top right**), simulation 3 (**bottom left**) and simulation 4 (**bottom right**).

Each simulation lasted 300 seconds. Each simulation (1)–(4) was repeated 100 times. Figure 12, Figure 13, Figure 14 and Figure 15 show the example layout before and after each simulation. Analyzing individual images, it is difficult to discern which layout has more disorder. For this reason, the entropy charts for individual simulations (Figure 16) are presented and the entropy values for all simulations are compared in Table 1.

It can be observed that the change in the arrangement of the system (creating the mess) increases the entropy of the simulated system, which translates into a reduction in the number of accesses to facilities by individual agents. In case the desired object is not in its original location, the agent searches for it until it is found. This action takes the agent’s time, so during the simulation, the agent can find fewer objects than in the case of an ordered system.

**Figure 17:**
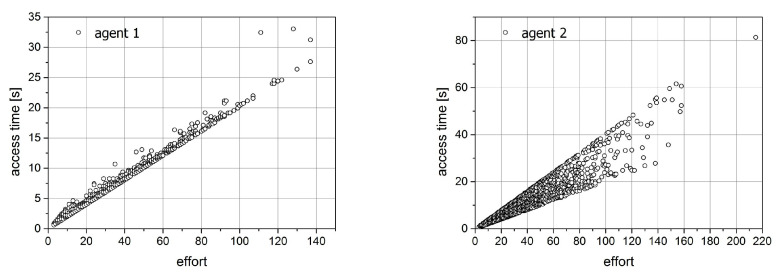
The relation between the effort and access time for simulation 1.

**Figure 18:**
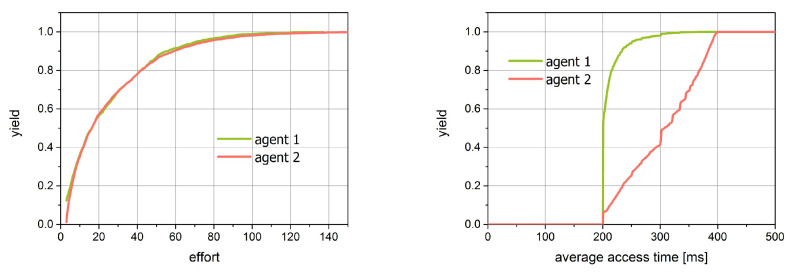
The cumulative distribution of effort and average single access for simulation 1.

Some interesting effects can be observed when analyzing the time characteristics of the state-change process. For all performed simulations, we prepared four plots that can be observed in Figure 17 and Figure 18 for the first simulation. The first pair of plots (Figure 17) shows the relation between the effort of the particular agent and the time spent on this effort. The crucial observation is that the probability of placing the collected item in a different place influences the apparent dependencies.

In the selected set of simulation parameters enlisted in the beginning of this section, certainly, the first simulation differs from another one due to the asymmetry of probabilities. This asymmetry is reflected in the shape of time versus effort plots (Figure 17). An interesting effect can be observed when analysing the access time versus effort plot. While for the second agent, whose activity is described by the probability 0.1, the points from simulation are concentrated along the straight line, for the first agent (probability 0.9), they are organized in the conic sector of plot with the higher values almost two times greater than the minimal one. Since such effect is not observed for other simulations, where the values of probabilities are equal we can connect it to the asymmetry of probabilities.

Figure 18 reflects the abovementioned effects. The distributions are presented here in two ways. In the left plot, there is a cumulative distribution of effort, while in the right one, there is the average access time. As seen, the distribution of effort measured in the number of attempts is exactly the same for both agents. In order to find some regularities we make some simple statistical analysis of these distributions. Indeed, we performed the regression calculations for the cumulative distributions of effort values. The results, shown in Table 2 in the form of correlation coefficient and the regression coefficient confirm the exponential character of these dependencies. However, significant differences are revealed in the right plot. With the different values of probabilities, not only quantitative differences exist but also qualitative ones. For Agent 1, we observe the same values as the efforts in the exponential distribution; however, for Agent 2, this distribution is in the interval (200, 400) ms and is almost uniform, leading to the linear run of the cumulative distribution.

**Figure 19:**
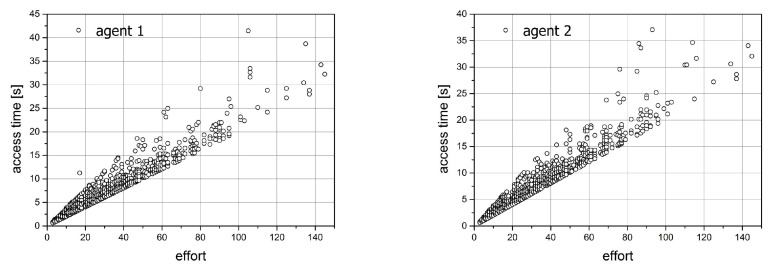
The relation between the effort and access time for simulation 2.

**Figure 20:**
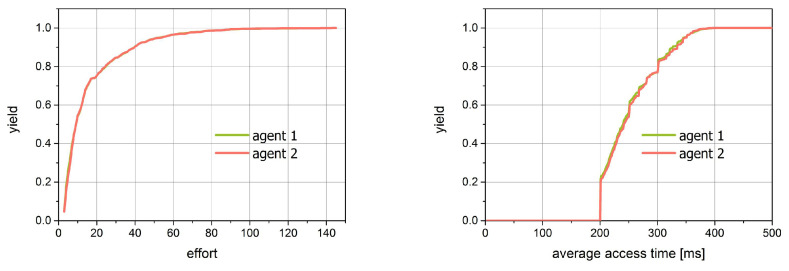
The cumulative distribution of effort and average single access for simulation 2.

For the second simulation, we prepared the same figures as those for simulation 1. They are shown in Figure 19 and Figure 20. Since both agents act here in the same way, we expect the compatibility of all characteristics. This fact is confirmed in the figures. The image of the access time versus effort dependencies looks identical for both distributions.

The distributions of effort as well as for the average access time are the exponential ones.

**Figure 21:**
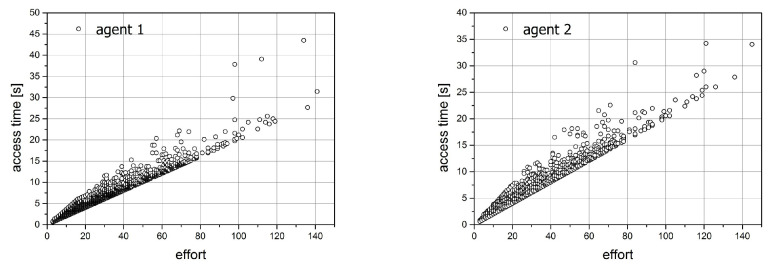
The relation between the effort and access time for simulation 3.

**Figure 22:**
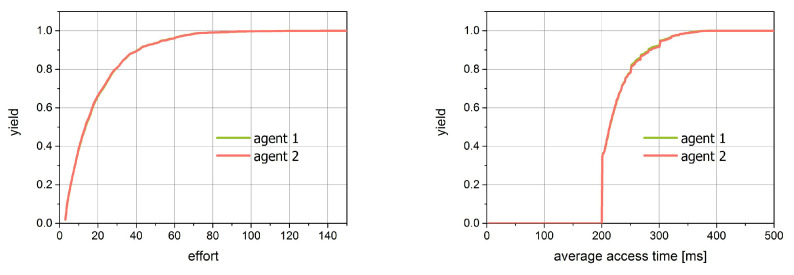
The cumulative distribution of effort and average single access for simulation 3.

For simulation 3, we repeat the determination of access characteristics, visible in Figure 21 and Figure 22. Let us here mention only briefly that, qualitatively, the result is the same as that in simulation 2. The plots in Figure 21 are indistinguishable one from another and the distributions in Figure 22 on both plots and for both agents can be described by the exponential distribution.

**Figure 23:**
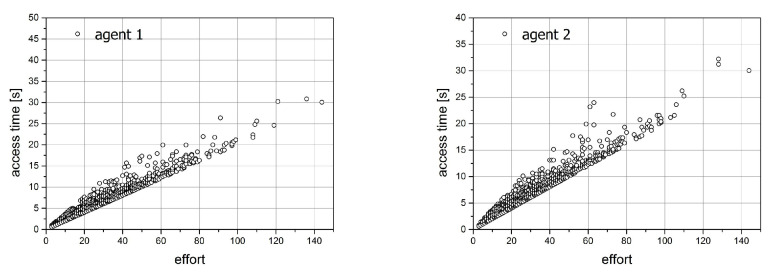
The relation between the effort and access time for simulation 4.

**Figure 24:**
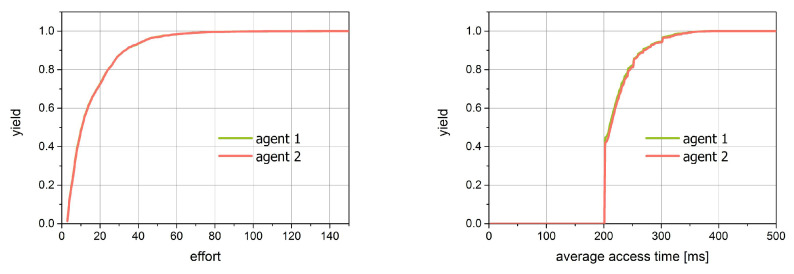
The cumulative distribution of effort and average single access for simulation 4.

Additionally, the access characteristics for simulation 4, presented in Figure 23 and Figure 24, follow the regularity observed earlier. It concerns the similarity of access time versus effort plots, as well as the exponential character of both distributions.

Table 2 presents the comparison of regression coefficients obtained for all 4 simulations. The successive columns present as follows:

- the lowest average attempt time—the linear regression coefficient calculated for the lowest line of attempt time versus effort plots.
- effort—the exponential regression coefficient for the distribution of effort. In the parentheses—the correlation coefficient.
- average attempt time—the exponential regression coefficients for the distribution of average attempt time. In the parentheses—the correlation coefficient.

The data in Table 2 show some similarities but also some deviations from the predicted order. When analyzing the second column related to the linear plots describing the simulations we can observe that, while other values are the same, the result for simulation 2 (both probabilities equal 0.1) shows the slope is higher by a factor of almost 20%. This effect cannot be explained with the small statistics since every plot was created for 100 simulation runs. The values in the third and fourth columns are calculated with the assumption that all distributions, for efforts as well as average attempt times, are the exponential ones. This assumption can be confirmed by the, mentioned earlier, regression analysis. Additionally, the result, which is not shown in the table, indicating that the description of the effort distribution that is exponential is the weakest (also taking into account the statistical description) among all presented cases leads to the conclusion that a more detailed analysis of the data is necessary.

This remark is confirmed by the value in the first row. The coefficients concerning the case with mixed probabilities {0.9,0.1} can be in no way explained by mixing the values for these parameters separately, which means that the system with different probabilities has significantly different dynamics than those of the uniform one.

## 5. Discussion

Let us start the recapitulation from some general remarks. It turns out to be a difficult task to study the order versus disorder relationship when these notions have to be related to some particular systems as their features. Certainly, we can think about the concept of order on several levels. For example, when looking at it etymologically, we can see the systematic, regular distribution or the arrangement of some objects or ideas (things, plans, intentions, etc.). It is unambiguously related to the proper harmony describing this system.

For us, the crucial scientific meaning lies in the three kinds of order that are usually defined as the mathematical one, the social one and the architectural one.

In the presented paper, we focused on the mathematical order, which is one of the original concepts of mathematical logic—the relationship between objects in a closed system. Such an ordering we contrast to chaos, which is understood as the stochastic behavior of deterministic physical systems [41].

Our closed system is limited when taking into account the number of elements and, following this fact, the number of states. The number of elements and states considered a mess (or disorder) depends on the assumed definition of what influences the detailed definition of entropy. We use the ABM as the supplemental mechanism for studying the effects of producing of a disorder, distinguishing the different modes of people’s behavior. That is why we present the different types of individuals’ behaviors by the different parameters of agents. The successive simulations are performed for the probabilities of creating disorder and are chosen from the set {0.1,0.5,0.9}.

The study shows that the model of entropy allows for the evaluation of the ordering state of a closed system. On the other hand, entropy is not the only measure that helps us to understand completely the results observed during the simulation. It turns out that the number of attempts when accessing particular objects can introduce considerable interesting information. We understand it as a trial to find an object that has to be found in some expected position. Let us show an example. During simulation 1, agent 1 makes, on average, approximately 700 trials to access the objects, while agent 2 makes approximately 50 trials.

Agent 2 tries to keep the order (places the object in a random place with probability 0.1) but his/her access is permanently obstructed. This is because agent 1 has a high probability (0.9) of not placing objects correctly. This fact is also confirmed by the average efforts: 2 and 145 for the agents 1 and 2, respectively. Some sudden changes for this value correspond to these iterations when agent 2 is forced to look for the object. The values of effort for agent 1 are low because he/she knows the current coordinates of the objects that were earlier rearranged by him/her. Therefore, only agent 2 need not search for it but agent 1 has no knowledge about the fact of the rearrangement.

For comparison, we can point out every other simulation where both agents put the objects in random places with the same probability. Both then have a comparable problem of finding objects.

During the numerous simulations, we notice that the increase in entropy explicitly causes the decrease in the number of accesses to the objects. With the maximum value of entropy, this number is as much as two times lower than that of the earlier accesses.

During the simulations, when the probability of random replacement is extremely high (0.9), the number of accesses is the lowest among all simulations, despite the fact that entropy does not reach the highest possible value (during the assumed simulation time). On the other hand, the entropy shows the fastest increase when at least one of the agents generates a mess with high probability.

We can also formulate some more general conclusions that may be well known from everyday life. With increasing disorder in some common space, shared by several persons, the time needed to localize the necessary object also increases but the mess-generating people are able to localize them faster.

The model and approach that are presented here in the initial version, have, in our opinion, large possibilities when used in a study of some theoretical models, like the resource assignment or some optimization mechanisms as well as in real social behavior (we think here especially on the inference processes and mutual relations between cooperating individuals, for example, house, children’s room, office, construction crew, project team). We are going to concentrate on the problem of different agents’ strategies, especially taking into account their purposefulness.

## 6. Conclusions

As it was mentioned in the introduction and related work, there is a large bibliography of papers related to the problem of disorder. Here, the explicit majority comes from the area of social and philosophical sciences. In our paper, we tried to present another aspect of the concept of disorder. We focused more on the mathematical description of the effects and consequences concerning the process of making the mess instead of its sources and psychological reasons. By using multiagent modeling, we showed the following:

- the measure of entropy is useful and appropriate when describing the disordering of the system;
- we should consider other measures together with entropy, for example, the effort or average number of attempts;
- the coexistence of different individuals with different tendencies to tidiness interferes more in the one more inclined to keep the order; and
- the time of ordering the system increases with the increasing time of making a mess.

We showed the correctness of the main idea discussed in the paper. The different modes of behavior of agents/individuals influence the organization of a closed system and it is possible to study this effect with the measurement of entropy and some additional proposed parameters.

Further work will focus on the inclusion of agents’ strategies taken from the game theory.

## Figures and Tables

**Table 1:** Entropy values of the simulated system for individual simulations.

Simulation Number	Probability of Agents A1 and A2	Number of Interactions	Max. Entropy	Average Entropy
1	A1[0.9], A2[0.1]	792	221.63	179.49
2	A1[0.1], A2[0.1]	882	122.26	72.74
3	A1[0.5], A2[0.5]	517	202.69	141.42
4	A1[0.9], A2[0.9]	439	212.19	162.57

**Table 2:** The regression coefficients for time analysis.

Simulation Number	Probability of Agents A1 and A2	Lowest Average Attempt Time [s]	Effort	Average Attempt Time [s]
1	A1[0.9], A2[0.1]	0.201	−0.0445 (0.999)	−0.0341 (0.982)
2	A1[0.1], A2[0.1]	0.236	−0.0511 (0.997)	−0.0152 (0.993)
3	A1[0.5], A2[0.5]	0.201	−0.0595 (0.997)	−0.0265 (0.992)
4	A1[0.9], A2[0.9]	0.201	−0.0672 (0.997)	−0.0297 (0.996)
